# Mitochondrial ADP/ATP Carrier in Dodecylphosphocholine Binds Cardiolipins with Non-native Affinity

**DOI:** 10.1016/j.bpj.2017.09.019

**Published:** 2017-10-20

**Authors:** François Dehez, Paul Schanda, Martin S. King, Edmund R.S. Kunji, Christophe Chipot

**Affiliations:** 1 Laboratoire International Associé Centre National de la Recherche Scientifique et University of Illinois at Urbana-Champaign, Unité Mixte de Recherche no. 7565, Université de Lorraine, Vandœuvre-lès-Nancy, France; 2 Université Grenoble Alpes, CEA, CNRS, Institut de Biologie Structurale, Grenoble, France; 3 Medical Research Council, Mitochondrial Biology Unit, University of Cambridge, Cambridge Biomedical Campus, Cambridge, United Kingdom; 4 Department of Physics, University of Illinois at Urbana-Champaign, Urbana, Illinois

## Abstract

Biophysical investigation of membrane proteins generally requires their extraction from native sources using detergents, a step that can lead, possibly irreversibly, to protein denaturation. The propensity of dodecylphosphocholine (DPC), a detergent widely utilized in NMR studies of membrane proteins, to distort their structure has been the subject of much controversy. It has been recently proposed that the binding specificity of the yeast mitochondrial ADP/ATP carrier (yAAC3) toward cardiolipins is preserved in DPC, thereby suggesting that DPC is a suitable environment in which to study membrane proteins. In this communication, we used all-atom molecular dynamics simulations to investigate the specific binding of cardiolipins to yAAC3. Our data demonstrate that the interaction interface observed in a native-like environment differs markedly from that inferred from an NMR investigation in DPC, implying that in this detergent, the protein structure is distorted. We further investigated yAAC3 solubilized in DPC and in the milder dodecylmaltoside with thermal-shift assays. The loss of thermal transition observed in DPC confirms that the protein is no longer properly folded in this environment.

## Main Text

**Figure 1:**
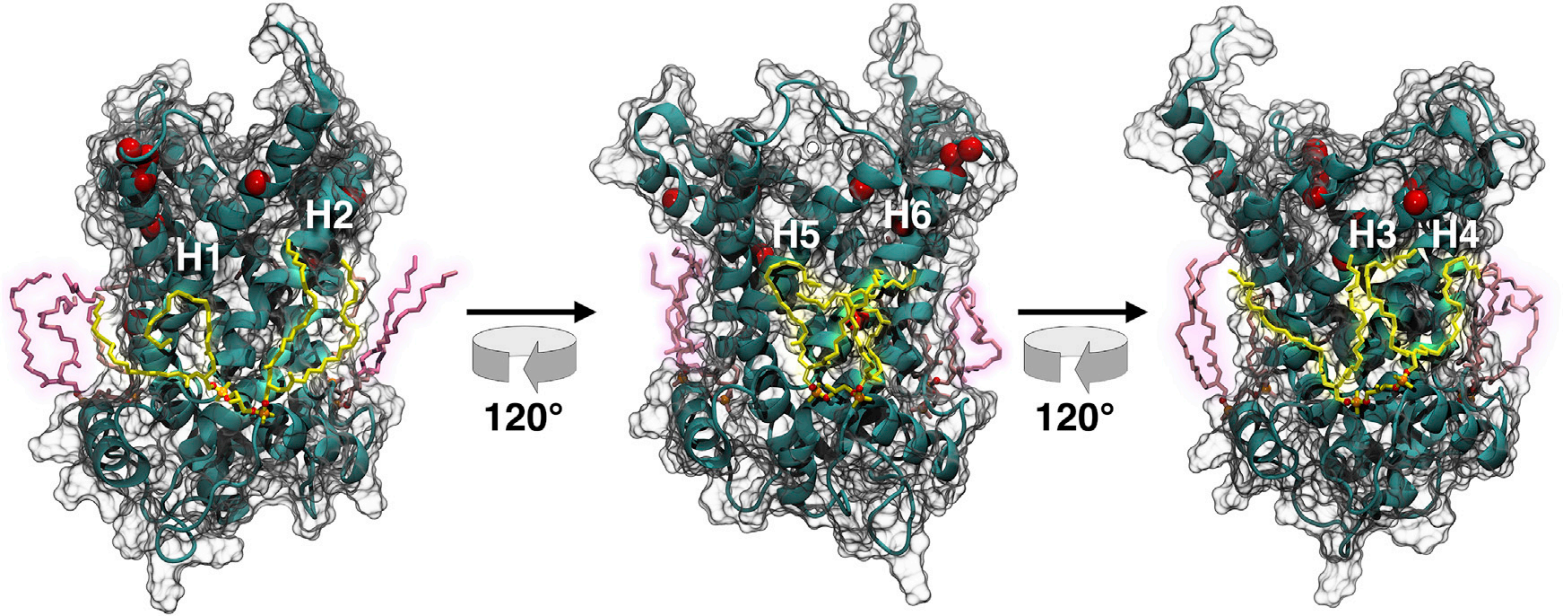
Snapshot of yAAC3 after ∼150 ns of a 200 ns MD simulation. The membrane carrier is represented as a transparent solvent-accessible surface with its secondary structure inside. The amide sites for which NOE crosspeaks to CL atoms were observed (16), namely, I14, I100, G118, G127, F220, L282, S292, Q298, M299, and I300, are highlighted as red van der Waals spheres. The three CLs binding yAAC3 are shown in yellow and pink to distinguish the region of the protein being examined. To see this figure in color, go online.

In the context of membrane-protein structure determination, dodecylphosphocholine (DPC) has been the subject of much criticism for being a very harsh detergent, prone to induce protein denaturation (1, 2, 3, 4, 5). Yet ∼40% of NMR investigations (6) have utilized this detergent, leading to protein structures (7, 8, 9) possessing a three-dimensional fold at variance with that observed in a different, milder environment (10, 11, 12, 13, 14). Among these membrane proteins, mitochondrial carriers have been the object of several studies (2, 3, 4). In particular, the uncoupling protein (UCP2), for which a backbone fold has been determined (8), and the ADP/ATP carrier (AAC), have been thoroughly investigated in thermostability-shift assay (TSA) experiments (4, 15). These experiments have shown that in a mild dodecylmaltoside (DDM) detergent environment, mitochondrial carriers extracted from native membranes show many of the expected features. Cooperative unfolding is observed, and addition of the inhibitor carboxyatractyloside (CATR) to AAC increases the stability and, hence, shifts the transition to a higher temperature. In contrast, when solubilized in DPC, the mitochondrial carrier UCP2 did not show any unfolding transition whatsoever (4), thereby strongly suggesting that in this harsh milieu, the protein is unfolded from the onset, or does not possess a stable tertiary structure. Transport assay experiments revealed that UCP2 purified in DPC (8) could not be resurrected into a functional state upon insertion into liposomes (3). Furthermore, molecular dynamics (MD) simulations indicated without any ambiguity that the putative structure of UCP2 determined by solution-state NMR in DPC (8), when inserted into membranes, collapses, suggesting that it is not a physiological state, and is possibly distorted beyond recovery by the harsh detergent environment. Recently, the functionality of the yeast mitochondrial AAC (yAAC3) has been investigated in DPC by looking at a very specific aspect of the carrier, namely, its ability to interact with cardiolipins (CLs) (16). CL is a highly abundant lipid in the inner mitochondrial membrane, and it has been shown to be important for the function of the transporter, albeit not strictly required (17). In the work of Zhao et al. (16), yAAC3 was prepared through a refolding procedure from inclusion bodies. The activity state was addressed indirectly by studying the well-characterized yAAC3-CL interactions observed in crystal structures extracted from the native membrane (13). Contacts of the protein with the surrounding CLs were identified by recording nuclear Overhauser enhancement (NOE) spectra. The NOE data characteristic of the CL headgroups are consistent with the crystallographic binding sites (13). These data further indicate close spatial proximity between the CL acyl chains and residues that are located at the cytoplasmic side of the carrier, i.e., far from the CL-headgroup binding sites (see Fig. 1). The main message of the work of Zhao et al. (16) is that DPC preserves the ability of AAC to bind CLs in a specific fashion, which implies that it is a good detergent, compatible with functional mitochondrial carriers. In this contribution, we turn to MD simulations to assess whether or not the binding specificity of CLs toward yAAC3 observed in DPC holds in a native-like membrane environment. Furthermore, we investigate by means of TSAs the ability of two detergents, namely, DPC and dodecylmaltoside (DDM), to preserve the fold of the mitochondrial carrier. In light of our findings, we propose that DPC alters the fold of AAC and modifies its interaction with CLs.

**Figure 2:**
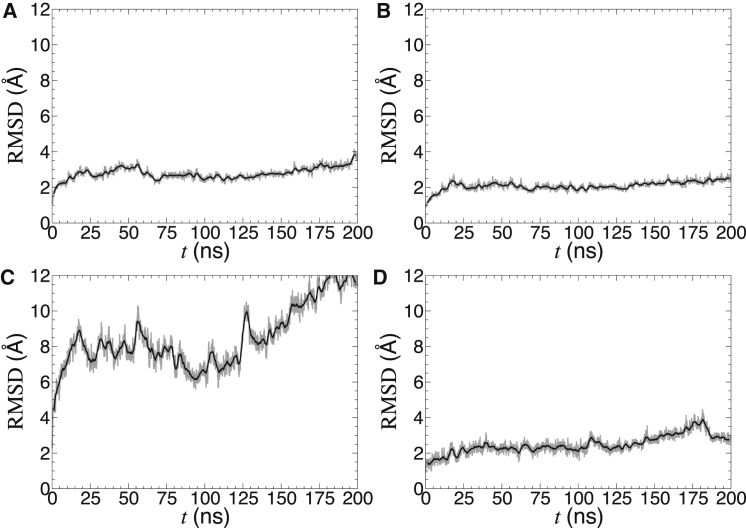
Distance root mean-square deviation (RMSD) from the starting configuration computed over the heavy atoms of yAAC3 (*A*), the *α*-helical content of yAAC3 (*B*), the acyl-chain heavy atoms of the three CLs binding the mitochondrial carrier (*C*), and the heavy atoms of the CL headgroups (*D*). The instantaneous values of the RMSD are shown in gray and the running average in black.

Starting from the crystal structure (13), we carried out an all-atom, 200-ns simulation of yAAC3 binding three CLs in a palmitoyloleoylphosphatidylcholine (POPC) bilayer (see the Supporting Material for methodological details). As can be observed in Fig. 2, the root mean-square deviation (RMSD) for yAAC3 averages to ∼3 Å for the entire protein, and to 2 Å for its secondary-structure elements, consistent with all other theoretical investigations of the mitochondrial AAC (18, 19, 20, 21). In addition, the alkyl chains of the three CLs are markedly disordered (see Fig. 1), with a substantial number of *trans*-*gauche* defects, congruent with the behavior in a CL-containing lipid bilayer (22). This disorder and mobility of the CLs is mirrored in the distance RMSDs of Fig. 2, which can be as large as 10 Å for the entire lipids, reduced to 3 Å for their headgroup, firmly attached to the membrane carrier between its three amphipathic helices. Consistent with the recent work of Hedger et al. (23), our simulation indicates that CLs seldom extend above the middle of the lipid bilayer, let alone to the cytoplasmic side, at variance with the NMR observations in DPC (16).

**Figure 3:**
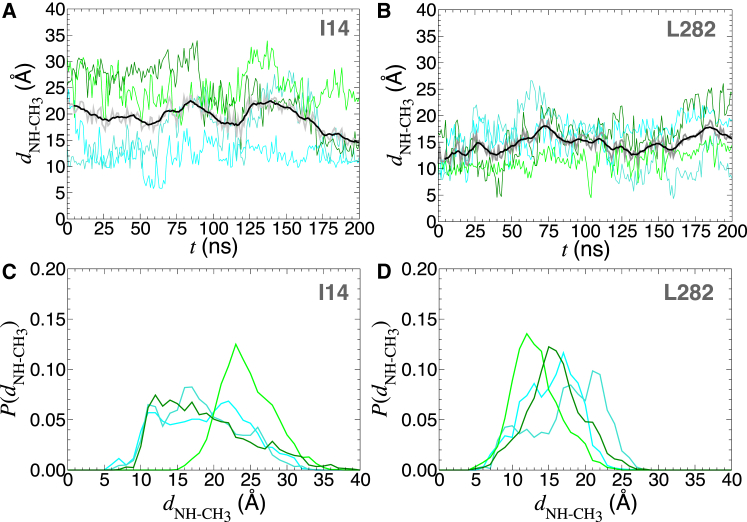
Distance separating the –NH hydrogen atom of residues I14 (*A*) and L282 (*B*) from the four terminal methyl groups of the closest CL. The four distances are shown as cyan, turquoise, light green, and dark green curves, alongside their mean, represented by a thick gray curve, and the running average thereof, represented as a black curve. Probability distributions of the four aforementioned hydrogen-methyl distances for residues I14 (*C*) and L282 (*D*). To see this figure in color, go online.

As can be seen in Fig. 3, for two residues involved in NOEs (16), namely, I14 and L282, lying not too far from the CLs, the distance separating the terminal methyl groups from the nitrogen atom of the amino acid can be appreciably large—typically >10 Å on average. More importantly, regardless of the residue, the range over which this distance is distributed exceeds 10 Å, thereby supporting the view of highly flexible alkyl chains and the absence of specific binding to the carrier (see the Supporting Material). From Figs. S2 and S3, it is clear that in a native-like membrane environment, the CL acyl chains cannot interact with the residues on the cytoplasmic side of the protein and hence are unlikely to give rise to NOEs, in stark contrast to observations in DPC. Our simulation demonstrates unambiguously that the CL acyl chains evolve far from many residues giving rise to NOE signals, hence suggesting that to satisfy the NOE constraints, the mitochondrial carrier must adopt a conformation in DPC distinct from the crystallographic one (10, 13). To ascertain that the dynamics of the CL acyl chains does not depend on the environment, we performed a separate MD simulation, wherein yAAC3 binding the three CLs is embedded in a DPC micelle (see Fig. S1). Just like in a POPC bilayer, the 100-ns trajectory reveals that the CL acyl chains extend too far from the residues involved in NOEs to rationalize the measured signals (16) (see the Supporting Material).

**Figure 4:**
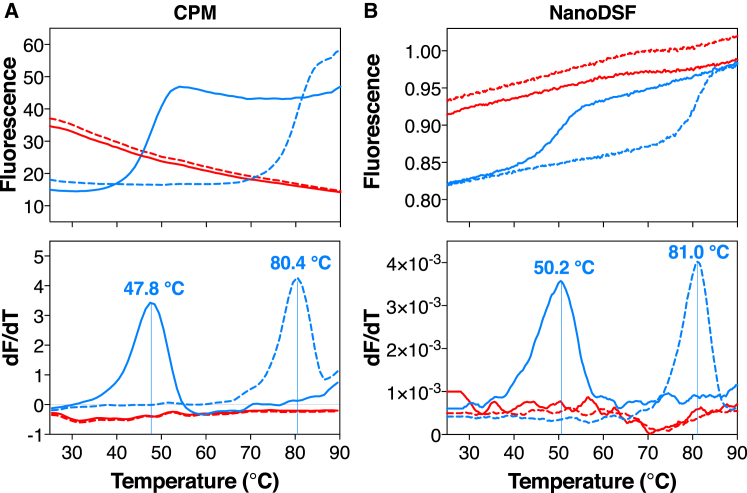
Thermostability of yAAC3 in 0.1% DDM (*blue line*) or 0.1% DPC (*red line*), in the presence (*dashed line*) and absence (*straight line*) of CATR. Thermostability was monitored using (*A*) the maleimide coumarin fluorophore CPM (24) and (*B*) nanoDSF (see Supporting Material). The top panels show the changes in fluorescence with temperature, whereas the bottom panels show the derivatives and the apparent melting temperatures, where they could be determined. To see this figure in color, go online.

To probe protein unfolding induced by DPC, we have applied two different thermal-shift assays (24) in DPC and DDM using 1) a maleimide coumarin fluorophore, 7-diethylamino-3-(4-maleimidophenyl)-4-methylcoumarin (CPM), and 2) dye-free differential scanning fluorimetry (nanoDSF) (see Supporting Material). As a control, we have added the specific inhibitor CATR, which brings the carrier in an aborted cytoplasmic state (13), leading to enhanced stability. Purified yAAC3 in DDM displayed a typical protein melting curve consistent with thermal denaturation of a folded protein, whether CPM or nanoDSF was used, giving apparent melting temperatures of 47.8 or 50.2°C, respectively (see Fig. 4). In the presence of CATR, a large shift in the melting temperature is observed, leading to apparent melting temperatures of 80.4 and 81.0°C, respectively. However, when the same yAAC3 preparation was diluted into DPC, high fluorescence signals at the start of the experiment were observed in both assays, showing no thermal transition, indicative of an unliganded AAC3 in an unfolded state. Addition of CATR did not alter the profiles, underscoring that yAAC3 in DPC has lost the ability to bind the inhibitor.

The apparent discrepancy between the reported experimental results (16) and the distances between the same atoms measured in our simulation, which are unlikely to produce detectable NOEs, calls into question the fold adopted by yAAC3 in DPC and the CL binding, which necessarily must be different from those in the crystal structures obtained from a native protein. There is a further hint for a different interaction in a native protein and in the refolded protein in DPC. In a native protein extracted from the membrane, CL molecules are very tightly bound, as they cannot be removed by extensive washes with detergent solutions (17, 25). The samples used for the crystal structures were obtained with yAAC3 purified from yeast mitochondria, and in this purification and crystallization protocol, no CL was present in the buffers, yet a clear density was discernible for the CL headgroups. This feature strongly suggests that the affinity of CL to native yAAC3 is very high. This property is shared with CATR, which binds with nanomolar affinity and also remains bound through the purification stages, though it is absent from the buffer. In such cases of high-affinity binding, addition of the binding partner, i.e., CLs, is expected to lead to disappearance of the NMR resonances corresponding to the free state and appearance of peaks characteristic of the bound state, which corresponds to a so-called slow-exchange regime. In sharp contrast, the NMR results in DPC reveal a clear signature of fast exchange upon addition of CLs, with gradually shifting crosspeaks and titration curves characteristic of low-affinity binding, often associated with nonspecific interactions, which might arise due to complementary charges on the protein and CL.

Binding of the inhibitor CATR to yAAC3 has also been shown to be distinct in DPC, compared to other environments, with a dissociation constant, $K_{\text{d}}$, of ∼150 *μ*M (26). However, this inhibitor is extremely toxic, and all studies published hitherto have shown that $K_{\text{d}}$ values fall within the low-nanomolar range (27, 28, 29, 30), in line with its toxicity, inhibiting the transporter in an aborted state. Interestingly enough, the most recent biochemical investigation reported a value of $K_{\text{d}}$ equal to 192 *μ*M (31). As it turns out, this surprising value was a typographical error, later corrected to 192 nM (32).

Taken together, our observations indicate that the binding features of CLs to yAAC3 witnessed in DPC are markedly distinct from those characteristic of a native-like membrane environment. Specifically, NOEs in DPC are not in agreement with the distance distributions observed from state-of-the-art simulations of the native structure embedded in a lipid bilayer. In fact, our simulation strongly suggests that to satisfy the NOE data, the mitochondrial carrier in DPC must be distorted. In other words, the interactions at play reflect nonspecific binding, likely to be driven by electrostatic interactions of the CL headgroups with an unfolded mitochondrial carrier. This claim is supported by TSAs, which, in the case of yAAC3 in DPC, reveals no temperature transition, in stark contrast with the same protein in the milder DDM environment.

## Author Contributions

F.D., P.S., E.R.S.K., and C.C. designed the research. F.D., P.S., M.S.K., E.R.S.K., and C.C. performed the research. F.D., P.S., E.R.S.K., and C.C. wrote the article; and all authors participated in the discussions and commented on the article.

## Supporting Material

Document S1. Supporting Materials and Methods and Figs. S1–S4

Document S2. Article plus Supporting Material
