# Supplementary material for: Mitochondrial ADP/ATP Carrier in Dodecylphosphocholine Binds Cardiolipins with Non-native Affinity
Source: Biophys J. 2017 Oct 20;113(11):2311–5. doi: 10.1016/j.bpj.2017.09.019 (PMC5722206; doi:10.1016/j.bpj.2017.09.019)
Supplement: Document S1. Supporting Materials and Methods and Figs. S1–S4 [file mmc1.pdf]

Biophysical Journal, Volume *113*

Supplemental Information

Mitochondrial ADP/ATP Carrier in Dodecylphosphocholine Binds Cardiolipins with Non-native Affinity

François Dehez, Paul Schanda, Martin S. King, Edmund R.S. Kunji, and Christophe Chipot

Mitochondrial ADP/ATP carrier in dodecylphosphocholine binds cardiolipins with non-native affinity

Supplementary material

François Dehez,[1] Paul Schanda,[2,*] Martin S. King,[3]
Edmund R. S. Kunji,[3] Christophe Chipot[1,4,*]

[1] Laboratoire International Associé Centre National de la Recherche Scientifique et University of Illinois at Urbana-Champaign, Unité Mixte de Recherche n°7565, Université de Lorraine, B.P. 70239, 54506 Vandœuvre-lè-Nancy cedex, France, [2] Institut de Biologie Structurale, CEA–CNRS–Université Grenoble Alpes, 71, avenue des Martyrs, C.S. 10090, 38044 Grenoble Cedex 9, France, [3] Medical Research Council, Mitochondrial Biology Unit, University of Cambridge, Cambridge Biomedical Campus, Wellcome Trust/MRC Building, Hills Road, Cambridge, CB2 0XY, United Kingdom, [4] Department of Physics, University of Illinois at Urbana-Champaign, 1110 West Green Street, Urbana, Illinois 61801

Thermal–shift assays. AAC3 was purified as described for the related yeast mitochondrial ADP/ATP carrier AAC2 (1). The purified AAC3 was diluted 20-fold in buffer containing 0.1% dodecyl maltoside (DDM) or 0.1% dodecylphosphocholine (DPC) and analyzed by thermal-shift assays, as described in reference 1. Protein unfolding is monitored with the maleimide coumarin fluorophore 7-diethylamino-3-(4'-maleimidylphenyl)-4-methylcoumarin (CPM). CPM reacts with protein thiols (4 cysteine residues in yAAC3) to give a fluorescent adduct, as cysteines become solvent-exposed due to denaturation of the protein. Thermal unfolding analysis was also performed using dye-free differential scanning fluorimetry (nanoDSF), which monitors the variations in fluorescence due to changes in the environment of 3 tryptophan and 11 tyrosine residues in yAAC3. Approximately 2 μg of protein at a concentration of 1.5 μM was added into a final volume of 10 μL buffer B (20 mM HEPES pH 8.0, 100 mM NaCl) containing either 0.1% of DDM with 0.1 mg/ml of tetraoleoyl cardiolipin, or 0.1% of DPC with 0.1 mg/ml tetraoleoyl cardiolipin, with or without 10 μM carboxyatractyloside, and the samples loaded into nanoDSF-grade standard glass capillaries. The temperature was increased by 5°C/min from 20 to 95°C, and the intrinsic fluorescence measured in a Prometheus NT.48 nanoDSF device (NanoTemper Technologies).

Simulation assays. For the simulation in a membrane mimetic, the molecular assembly consisted of yeast mitochondrial ADP/ ATP carrier yAAC3 embedded in a pre–equilibrated patch of 1–palmitoyl–2–oleo-yl–*sn*–glycero–3–phosphatidylcholine (POPC) with 18:2,18:2–cardiolipins (CLs), specifically tetralinoleyl–*sn*–glycero–*bis*–3–diphosphatidylglycerol (TLCL), consistent with the choice of lipids of Zhao et al. (2). Use was made of the crystallographic structure of yAAC3 obtained by Ruprecht et al. (3). Missing elements, in particular the N–terminus residues and one loop, were inferred using the program MODELLER (4). The lipid bilayer was formed by 124 POPC units and three CLs hydrated by 10,904 TIP3P(5) water molecules. After proper equilibration, the dimensions of the assay were approximately 74 $\times$ 74 $\times$ 98 Å^3. The molecular dynamics (MD) simulations were performed with a salinity of 0.15 M NaCl. The simulation assay was constructed employing the CHARMM–GUI membrane builder (6), which supplies randomized acyl–chain conformations for all lipid components. For the simulation in a micellar environment, yAAC3 binding the three CLs was embedded in DPC micelle with a detergent concentration of 300 nM, and a protein:detergent ratio of 1:250. After proper equilibration, the dimensions of the assay were approximately 103 $\times$ 103 $\times$ 103 Å^3. The all–atom CHARMM C36 force field (7) was used to describe water, yAAC3, the lipid bilayer (8) and the ions.

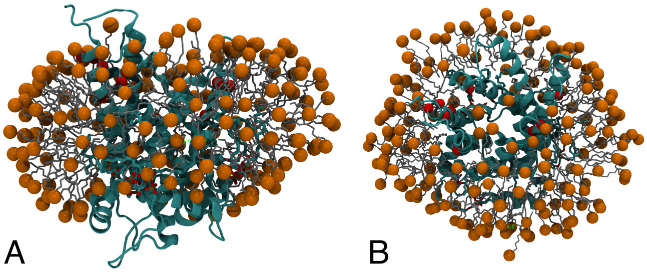

FIGURE S1: yAAC3 binding CLs embedded in a DPC micelle at a detergent concentration of 300 nM. (A) Side view and (B) top view.

© 2016 Biophysical Society
0006-3495/08/09/2624/12 $2.00

doi:

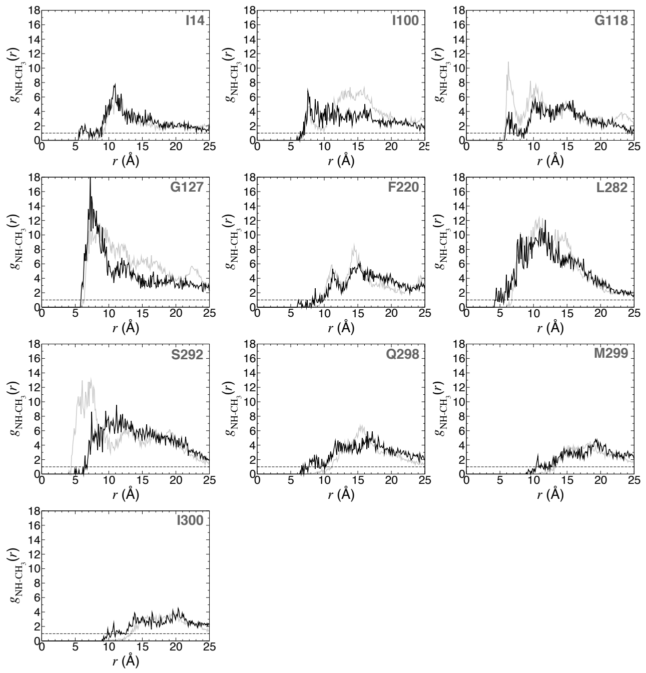

FIGURE S2: **Pair correlation functions between the –NH hydrogen atom of I14, I100, G118, G127, F220, L282, S292, Q298, M299 and I300, for which NOE effects were recorded by Zhao et al. (2) and the four terminal methyl groups of the three CLs bound to the mitochondrial carrier. The pair correlation functions were inferred from simulations in a POPC bilayer (dark lines) and in a DPC micelle (light lines).**

Molecular dynamics simulations. All simulations were performed in the isobaric–isothermal ensemble, using the scalable MD program NAMD 2.12 (9). Consistent with Zhao et al. (2), The temperature and the pressure were maintained at 300 K and 1 atm, respectively, using Langevin dynamics and the Langevin piston method, with anisotropic scaling of the simulation cell for the POPC assay (10). The r–RESPA multiple time–step algorithm(11) was employed to integrate the equations of motion with a time step of 1 and 2 fs for short– and long–range interactions, respectively. Covalent bonds in yAAC3 and in the lipids involving hydrogen atoms were constrained to their equilibrium length by means of the SHAKE/RATTLE algorithms (12, 13), and the SETTLE algorithms (14) for water. Long–range electrostatic forces were taken into account by means of the particle mesh Ewald algorithm (15) A 12–Å cutoff was introduced to truncate van der Waals and short–range Coulombic interactions. A switching function was introduced for van der Waals forces (8). Periodic boundary conditions were applied in the three directions of Cartesian space. The simulation protocols consisted of the following steps. For yAAC3 in a POPC bilayer, (i) after suitable energy minimization, the simulation assay was thermalized over 10.0 ns, during which both the protein and the CL head groups were harmonically tethered to their initial position, thereby allowing the lipid acyl chains to relax around the mitochondrial carrier. (ii) The assay was submitted to an additional 10.0–ns equilibration step, during which the positional harmonic restraints were removed and the entire molecular assembly was free to relax. (iii) A 200.0–ns production run was performed, from which configurations were stored every 10 ps for analysis purposes. For yAAC3 in a micellar environment, (i) after suitable energy minimization, the simulation assay was thermalized over 50.0 ns, during which both the protein and the CL head groups were harmonically tethered to their initial position, allowing the DPC units to optimize their interaction with the membrane carrier. (ii) A 100–ns production run was performed, from which configurations were stored every 10 ps for analysis purposes. Visualization and analyses of the MD trajectories were performed with the VMD program.(16)

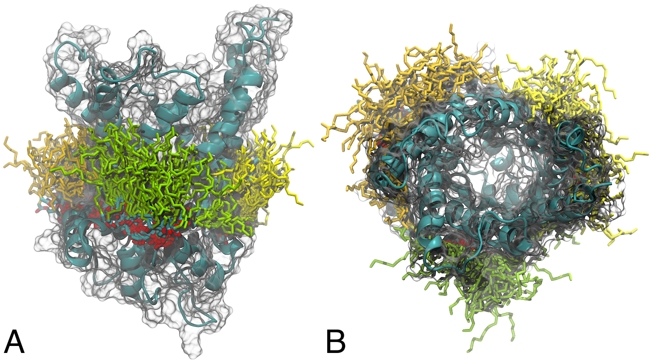

FIGURE S3: **Overlay onto the yAAC3 structure of CL conformations extracted every 10 ns from the 200–ns MD trajectory. (A) Side view and (B) top view. The superimposition of different conformations of the CLs underscores that while the head-group region remains tightly bound to the mitochondrial carrier in a specific fashion, the position of the acyl chains fluctuates markedly, indicative of a lack of specificity towards the protein. This representation further reinforces the idea that the CL acyl chains are highly unlikely to span the two leaflets of the lipid bilayer to interact with residues lining the mouth of the carrier, e.g., I300.**

NOE distances. In addition to the series of distances measured between the nitrogen atom of two amino acids, namely I14 and L282, and the terminal methyl group of the three CLs, and reported in the main text, a more systematic analysis was performed, wherein pair correlation functions (17) were determined for all the residues reported by Zhao et al. (see Figure S4 of reference 2), i.e., I14, I100, G118, G127, F220, L282, S292, Q298, M299 and I300. As can be seen in Figure S2, the computed pair correlation functions are strikingly at variance with the distances of Zhao et al. Consistent with Figure 3 of the main text, they reflect disorder and mobility of the CL acyl chains, as can be expected from thermal motion at 310 K, and the virtual impossibility of specific binding motifs with the mitochondrial carrier (see Figure S3).

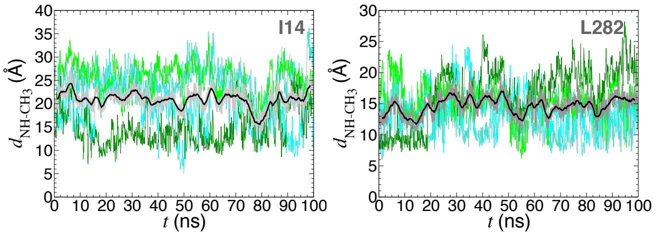

FIGURE S4: Distance separating the –NH hydrogen atom of residues I14 (left) and L282 (right) from the four terminal methyl group of the closest CL, as measured from the DPC assay. The four distances are shown as cyan, turquoise, light and dark green curves, alongside their mean as a thick grey curve and running average thereof as a black curve.

References

1. Crichton, P. G., Lee, Y., Ruprecht, J. J., Cerson, E., Thangaratnarajah, C., King, M. S., and Kunji, E. R. S. (2015) Trends in thermostability provide information on the nature of substrate, inhibitor, and lipid interactions with mitochondrial carriers. *J. Biol. Chem. 290*, 8206–8217.
2. Zhao, L., Wang, S., Run, C., OuYang, B., and Chou, J. J. (2016) Specific lipid binding of membrane proteins in detergent micelles characterized by NMR and molecular dynamics. *Biochemistry 55*, 5317–5320.
3. Ruprecht, J. J., Hellawell, A. M., Harding, M., Crichton, P. G., McCoy, A. J., and Kunji, E. R. S. (2014) Structures of yeast mitochondrial ADP/ATP carriers support a domain-based alternating–access transport mechanism. *Proc. Natl. Acad. Sci. U.S.A. 111*, E426–E434.
4. Webb, B., and Săli, A. (2014) Protein structure modeling with MODELLER. *Met. Mol. Biol.(Clifton, N.J.) 1137*, 1–15.
5. Jorgensen, W. L., Chandrasekhar, J., Madura, J. D., Impey, R. W., and Klein, M. L. (1983) Comparison of simple potential functions for simulating liquid water. *J. Chem. Phys. 79*, 926–935.
6. Jo, S., Lim, J. B., Klauda, J. B., and Im, W. (2009) CHARMM-GUI Membrane Builder for mixed bilayers and its application to yeast membranes. *Biophys. J. 97*, 50–58.
7. MacKerell Jr., A. D. et al. (1998) All–atom empirical potential for molecular modeling and dynamics studies of proteins. *J. Phys. Chem. B 102*, 3586–3616.
8. Klauda, J. B., Venable, R. M., Freites, J. A., O'Connor, J. W., Tobias, D. J., Mondragon-Ramirez, C., Vorobyov, I., MacKerell, A. D., Jr, and Pastor, R. W. (2010) Update of the CHARMM all–atom additive force field for lipids: Validation on six lipid types. *J. Phys. Chem. B 114*, 7830–7843.
9. Phillips, J. C., Braun, R., Wang, W., Gumbart, J., Tajkhorshid, E., Villa, E., Chipot, C., Skeel, L., R. D. Kalé, and Schulten, K. (2005) Scalable molecular dynamics with NAMD. *J. Comput. Chem. 26*, 1781–1802.
10. Feller, S. E., Zhang, Y. H., Pastor, R. W., and Brooks, B. R. (1995) Constant pressure molecular dynamics simulations — The Langevin piston method. *J. Chem. Phys. 103*, 4613–4621.
11. Tuckerman, M. E., Berne, B. J., and Martyna, G. J. (1992) Reversible multiple time scale molecular dynamics. *J. Phys. Chem. B 97*, 1990–2001.
12. Ryckaert, J., Ciccotti, G., and Berendsen, H. J. C. (1977) Numerical integration of the Cartesian equations of motion for a system with constraints: Molecular dynamics of n–alkanes. *J. Comput. Phys. 23*, 327–341.
13. Andersen, H. C. (1983) Rattle: a "velocity" version of the shake algorithm for molecular dynamics calculations. *J. Comput. Phys. 52*, 24–34.
14. Miyamoto, S., and Kollman, P. A. (1992) SETTLE: An analytical version of the SHAKE and RATTLE algorithms for rigid water models. *J. Comput. Chem. 13*, 952–962.
15. Darden, T. A., York, D. M., and Pedersen, L. G. (1993) Particle mesh Ewald: An $N log N$ method for ewald sums in large systems. *J. Chem. Phys. 98*, 10089–10092.
16. Humphrey, W., Dalke, A., and Schulten, K. (1996) VMD — Visual molecular dynamics. *J. Molec. Graphics 14*, 33–38.
17. Chandler, D. *Introduction to modern statistical mechanics*; Oxford University Press, 1987.
