# Supplementary material for: Mitochondrial ADP/ATP Carrier in Dodecylphosphocholine Binds Cardiolipins with Non-native Affinity
Source: Biophys J. 2017 Oct 20;113(11):2311–5. doi: 10.1016/j.bpj.2017.09.019 (PMC5722206; doi:10.1016/j.bpj.2017.09.019)
Supplement: Document S2. Article plus Supporting Material [file mmc2.pdf]

Biophysical Journal
Biophysical Letter

Biophysical Society

Mitochondrial ADP/ATP Carrier in Dodecylphosphocholine Binds Cardiolipins with Non-native Affinity

François Dehez,[1] Paul Schanda,[2,*] Martin S. King,[3] Edmund R. S. Kunji,[3] and Christophe Chipot[1,4,*]
[1]Laboratoire International Associé Centre National de la Recherche Scientifique et University of Illinois at Urbana-Champaign, Unité Mixte de Recherche no. 7565, Université de Lorraine, Vandœuvre-lès-Nancy, France; [2]Université Grenoble Alpes, CEA, CNRS, Institut de Biologie Structurale, Grenoble, France; [3]Medical Research Council, Mitochondrial Biology Unit, University of Cambridge, Cambridge Biomedical Campus, Cambridge, United Kingdom; and [4]Department of Physics, University of Illinois at Urbana-Champaign, Urbana, Illinois

ABSTRACT Biophysical investigation of membrane proteins generally requires their extraction from native sources using detergents, a step that can lead, possibly irreversibly, to protein denaturation. The propensity of dodecylphosphocholine (DPC), a detergent widely utilized in NMR studies of membrane proteins, to distort their structure has been the subject of much controversy. It has been recently proposed that the binding specificity of the yeast mitochondrial ADP/ATP carrier (yAAC3) toward cardiolipins is preserved in DPC, thereby suggesting that DPC is a suitable environment in which to study membrane proteins. In this communication, we used all-atom molecular dynamics simulations to investigate the specific binding of cardiolipins to yAAC3. Our data demonstrate that the interaction interface observed in a native-like environment differs markedly from that inferred from an NMR investigation in DPC, implying that in this detergent, the protein structure is distorted. We further investigated yAAC3 solubilized in DPC and in the milder dodecylmaltoside with thermal-shift assays. The loss of thermal transition observed in DPC confirms that the protein is no longer properly folded in this environment.

In the context of membrane-protein structure determination, dodecylphosphocholine (DPC) has been the subject of much criticism for being a very harsh detergent, prone to induce protein denaturation (1–5). Yet ∼40% of NMR investigations (6) have utilized this detergent, leading to protein structures (7–9) possessing a three-dimensional fold at variance with that observed in a different, milder environment (10–14). Among these membrane proteins, mitochondrial carriers have been the object of several studies (2–4). In particular, the uncoupling protein (UCP2), for which a backbone fold has been determined (8), and the ADP/ATP carrier (AAC), have been thoroughly investigated in thermostability-shift assay (TSA) experiments (4,15). These experiments have shown that in a mild dodecylmaltoside (DDM) detergent environment, mitochondrial carriers extracted from native membranes show many of the expected features. Cooperative unfolding is observed, and addition of the inhibitor carboxyatractyloside (CATR) to AAC increases the stability and, hence, shifts the transition to a higher temperature. In contrast, when solubilized in DPC, the mitochondrial carrier UCP2 did not show any unfolding transition whatsoever (4), thereby strongly suggesting that in this harsh milieu, the protein is unfolded from the onset, or does not possess a stable tertiary structure. Transport assay experiments revealed that UCP2 purified in DPC (8) could not be resurrected into a functional state upon insertion into liposomes (3). Furthermore, molecular dynamics (MD) simulations indicated without any ambiguity that the putative structure of UCP2 determined by solution-state NMR in DPC (8), when inserted into membranes, collapses, suggesting that it is not a physiological state, and is possibly distorted beyond recovery by the harsh detergent environment. Recently, the functionality of the yeast mitochondrial AAC (yAAC3) has been investigated in DPC by looking at a very specific aspect of the carrier, namely, its ability to interact with cardiolipins (CLs) (16). CL is a highly abundant lipid in the inner mitochondrial membrane, and it has been shown to be important for the function of the transporter, albeit not strictly required (17). In the work of Zhao et al. (16), yAAC3 was prepared through a refolding procedure from inclusion bodies. The activity state was addressed indirectly by studying the well-characterized yAAC3-CL interactions observed in crystal structures extracted from the native membrane (13). Contacts of the protein with the surrounding CLs were identified by recording

Submitted July 7, 2017, and accepted for publication September 21, 2017.
*Correspondence: paul.schanda@ibs.fr or chipot@ks.uiuc.edu
Editor: Jose Faraldo-Gomez.
https://doi.org/10.1016/j.bpj.2017.09.019
© 2017 Biophysical Society.
This is an open access article under the CC BY license (http://creativecommons.org/licenses/by/4.0/).
CrossMark

nuclear Overhauser enhancement (NOE) spectra. The NOE data characteristic of the CL headgroups are consistent with the crystallographic binding sites (13). These data further indicate close spatial proximity between the CL acyl chains and residues that are located at the cytoplasmic side of the carrier, i.e., far from the CL-headgroup binding sites (see Fig. 1). The main message of the work of Zhao et al. (16) is that DPC preserves the ability of AAC to bind CLs in a specific fashion, which implies that it is a good detergent, compatible with functional mitochondrial carriers. In this contribution, we turn to MD simulations to assess whether or not the binding specificity of CLs toward yAAC3 observed in DPC holds in a native-like membrane environment. Furthermore, we investigate by means of TSAs the ability of two detergents, namely, DPC and dodecylmaltoside (DDM), to preserve the fold of the mitochondrial carrier. In light of our findings, we propose that DPC alters the fold of AAC and modifies its interaction with CLs.

Starting from the crystal structure (13), we carried out an all-atom, 200-ns simulation of yAAC3 binding three CLs in a palmitoyloleoylphosphatidylcholine (POPC) bilayer (see the Supporting Material for methodological details). As can be observed in Fig. 2, the root mean-square deviation (RMSD) for yAAC3 averages to ~3 Å for the entire protein, and to 2 Å for its secondary-structure elements, consistent with all other theoretical investigations of the mitochondrial AAC (18–21). In addition, the alkyl chains of the three CLs are markedly disordered (see Fig. 1), with a substantial number of *trans-gauche* defects, congruent with the behavior in a CL-containing lipid bilayer (22). This disorder and mobility of the CLs is mirrored in the distance RMSDs of Fig. 2, which can be as large as 10 Å for the entire lipids, reduced to 3 Å for their headgroup, firmly attached to the membrane carrier between its three amphipathic helices. Consistent with the recent work of Hedger et al. (23), our simulation indicates that CLs seldom extend above the middle of the lipid bilayer, let alone to the cytoplasmic side, at variance with the NMR observations in DPC (16).

As can be seen in Fig. 3, for two residues involved in NOEs (16), namely, I14 and L282, lying not too far from the CLs, the distance separating the terminal methyl groups from the nitrogen atom of the amino acid can be appreciably large—typically >10 Å on average. More importantly, regardless of the residue, the range over which this distance is distributed exceeds 10 Å, thereby supporting the view of highly flexible alkyl chains and the absence of specific binding to the carrier (see the Supporting Material). From Figs. S2 and S3, it is clear that in a native-like membrane environment, the CL acyl chains cannot interact with the residues on the cytoplasmic side of the protein and hence are unlikely to give rise to NOEs, in stark contrast to observations in DPC. Our simulation demonstrates unambiguously that the CL acyl chains evolve far from many residues giving rise to NOE signals, hence suggesting that to satisfy the NOE constraints, the mitochondrial carrier must adopt a conformation in DPC distinct from the crystallographic one (10,13). To ascertain that the dynamics of the CL acyl chains does not depend on the environment, we performed a separate MD simulation, wherein yAAC3 binding the three CLs is embedded in a DPC micelle (see Fig. S1). Just like in a POPC bilayer, the 100-ns trajectory reveals that the CL acyl chains extend too far from the residues involved in NOEs to rationalize the measured signals (16) (see the Supporting Material).

To probe protein unfolding induced by DPC, we have applied two different thermal-shift assays (24) in DPC and DDM using 1) a maleimide coumarin fluorophore, 7-diethylamino-3-(4-maleimidophenyl)-4-methylcoumarin (CPM), and 2) dye-free differential scanning fluorimetry (nanoDSF) (see Supporting Material). As a control, we have added the specific inhibitor CATR, which brings the carrier in an

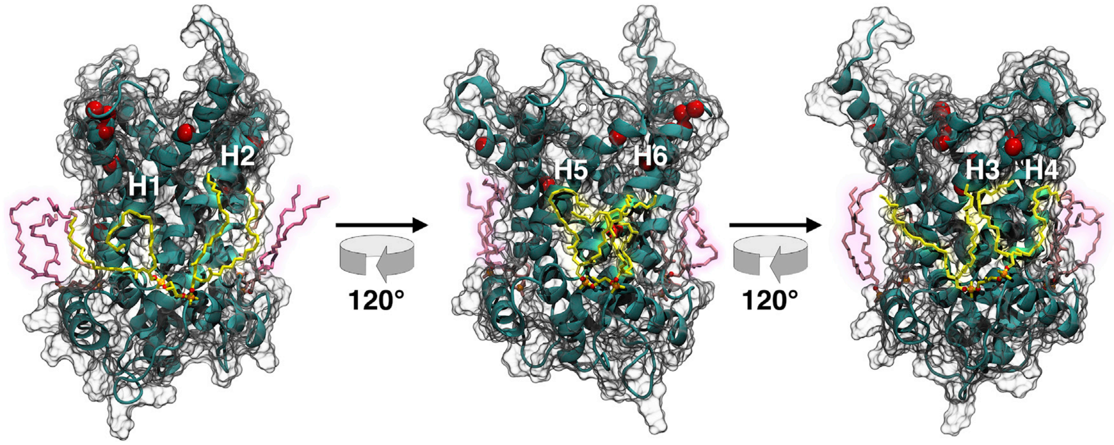

FIGURE 1 Snapshot of yAAC3 after ~150 ns of a 200 ns MD simulation. The membrane carrier is represented as a transparent solvent-accessible surface with its secondary structure inside. The amide sites for which NOE crosspeaks to CL atoms were observed (16), namely, I14, I100, G118, G127, F220, L282, S292, Q298, M299, and I300, are highlighted as red van der Waals spheres. The three CLs binding yAAC3 are shown in yellow and pink to distinguish the region of the protein being examined. To see this figure in color, go online.

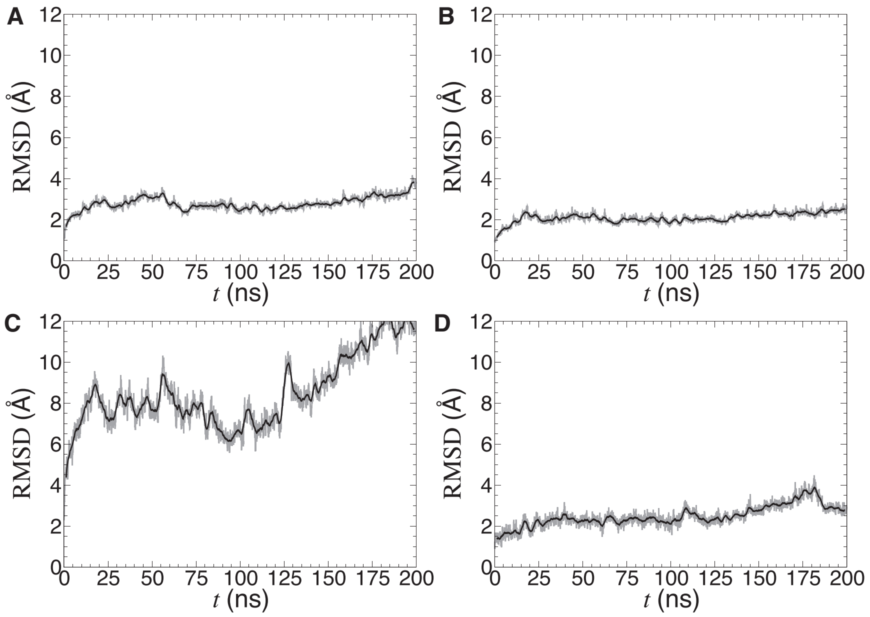

FIGURE 2 **Distance root mean-square deviation (RMSD) from the starting configuration computed over the heavy atoms of yAAC3 (*A*), the α-helical content of yAAC3 (*B*), the acyl-chain heavy atoms of the three CLs binding the mitochondrial carrier (*C*), and the heavy atoms of the CL headgroups (*D*). The instantaneous values of the RMSD are shown in gray and the running average in black.**

aborted cytoplasmic state (13), leading to enhanced stability. Purified yAAC3 in DDM displayed a typical protein melting curve consistent with thermal denaturation of a folded protein, whether CPM or nanoDSF was used, giving apparent melting temperatures of 47.8 or 50.2°C, respectively (see Fig. 4). In the presence of CATR, a large shift in the melting temperature is observed, leading to apparent melting temperatures of 80.4 and 81.0°C, respectively. However, when the same yAAC3 preparation was diluted into DPC, high fluorescence signals at the start of the experiment were observed in both assays, showing no thermal transition, indicative of an unliganded AAC3 in an unfolded state. Addition of CATR did not alter the profiles, underscoring that yAAC3 in DPC has lost the ability to bind the inhibitor.

The apparent discrepancy between the reported experimental results (16) and the distances between the same atoms measured in our simulation, which are unlikely to produce detectable NOEs, calls into question the fold adopted by yAAC3 in DPC and the CL binding, which necessarily must be different from those in the crystal structures obtained from a native protein. There is a further hint for a different interaction in a native protein and in the refolded protein in DPC. In a native protein extracted from the

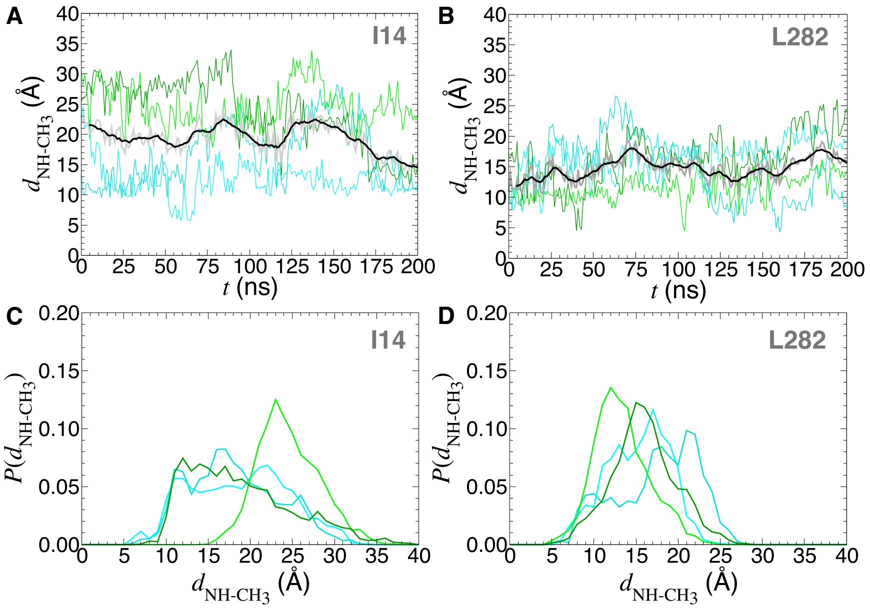

FIGURE 3 **Distance separating the –NH hydrogen atom of residues I14 (*A*) and L282 (*B*) from the four terminal methyl groups of the closest CL. The four distances are shown as cyan, turquoise, light green, and dark green curves, alongside their mean, represented by a thick gray curve, and the running average thereof, represented as a black curve. Probability distributions of the four aforementioned hydrogen-methyl distances for residues I14 (*C*) and L282 (*D*). To see this figure in color, go online.**

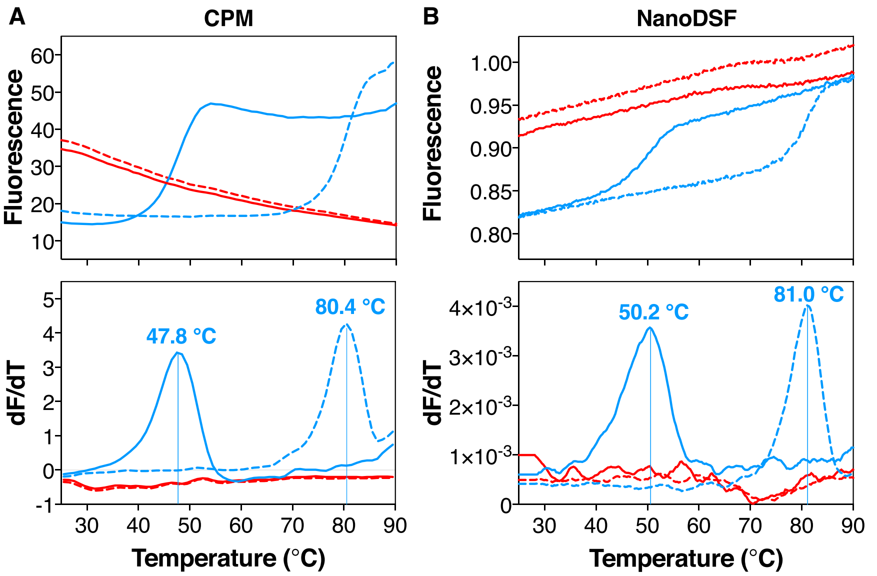

FIGURE 4 Thermostability of yAAC3 in 0.1% DDM (*blue line*) or 0.1% DPC (*red line*), in the presence (*dashed line*) and absence (*straight line*) of CATR. Thermostability was monitored using (*A*) the maleimide coumarin fluorophore CPM (24) and (*B*) nanoDSF (see Supporting Material). The top panels show the changes in fluorescence with temperature, whereas the bottom panels show the derivatives and the apparent melting temperatures, where they could be determined. To see this figure in color, go online.

membrane, CL molecules are very tightly bound, as they cannot be removed by extensive washes with detergent solutions (17,25). The samples used for the crystal structures were obtained with yAAC3 purified from yeast mitochondria, and in this purification and crystallization protocol, no CL was present in the buffers, yet a clear density was discernible for the CL headgroups. This feature strongly suggests that the affinity of CL to native yAAC3 is very high. This property is shared with CATR, which binds with nanomolar affinity and also remains bound through the purification stages, though it is absent from the buffer. In such cases of high-affinity binding, addition of the binding partner, i.e., CLs, is expected to lead to disappearance of the NMR resonances corresponding to the free state and appearance of peaks characteristic of the bound state, which corresponds to a so-called slow-exchange regime. In sharp contrast, the NMR results in DPC reveal a clear signature of fast exchange upon addition of CLs, with gradually shifting crosspeaks and titration curves characteristic of low-affinity binding, often associated with nonspecific interactions, which might arise due to complementary charges on the protein and CL.

Binding of the inhibitor CATR to yAAC3 has also been shown to be distinct in DPC, compared to other environments, with a dissociation constant, K_d, of $\sim$150 μM (26). However, this inhibitor is extremely toxic, and all studies published hitherto have shown that K_d values fall within the low-nanomolar range (27–30), in line with its toxicity, inhibiting the transporter in an aborted state. Interestingly enough, the most recent biochemical investigation reported a value of K_d equal to 192 μM (31). As it turns out, this surprising value was a typographical error, later corrected to 192 nM (32).

Taken together, our observations indicate that the binding features of CLs to yAAC3 witnessed in DPC are markedly distinct from those characteristic of a native-like membrane environment. Specifically, NOEs in DPC are not in agreement with the distance distributions observed from state-of-the-art simulations of the native structure embedded in a lipid bilayer. In fact, our simulation strongly suggests that to satisfy the NOE data, the mitochondrial carrier in DPC must be distorted. In other words, the interactions at play reflect nonspecific binding, likely to be driven by electrostatic interactions of the CL headgroups with an unfolded mitochondrial carrier. This claim is supported by TSAs, which, in the case of yAAC3 in DPC, reveals no temperature transition, in stark contrast with the same protein in the milder DDM environment.

SUPPORTING MATERIAL

Supporting Materials and Methods and four figures are available at http://www.biophysj.org/biophysj/supplemental/S0006-3495(17)31031-7.

AUTHOR CONTRIBUTIONS

F.D., P.S., E.R.S.K., and C.C. designed the research. F.D., P.S., M.S.K., E.R.S.K., and C.C. performed the research. F.D., P.S., E.R.S.K., and C.C. wrote the article; and all authors participated in the discussions and commented on the article.

ACKNOWLEDGMENTS

The authors gratefully acknowledge the Laboratoire International Associé Centre National de la Recherche Scientifique and University of Illinois at Urbana-Champaign. They are indebted to the Grand Équipement National de Calcul Intensif (GENCI) and the Centre Informatique National de l'Enseignement Supérieur (CINES) for generous provision of computer time. This work was supported by the European Research Council (ERC-StG-2012-311318-ProtDyn2Function) to P.S., and by program U105663139 of the UK Medical Research Council to M.S.K. and E.R.S.K.

REFERENCES

1. Maffeo, C., and A. Aksimentiev. 2009. Structure, dynamics, and ion conductance of the phospholamban pentamer. *Biophys. J.* 96:4853–4865.
2. Zhou, H. X., and T. A. Cross. 2013. Influences of membrane mimetic environments on membrane protein structures. *Annu. Rev. Biophys.* 42:361–392.
3. Zoonens, M., S. Masscheleyn, …, F. Dehez. 2013. Dangerous liaisons between detergents and membrane proteins. The case of mitochondrial uncoupling protein 2. *J. Am. Chem. Soc.* 135:15174–15182.
4. Crichton, P. G., Y. Lee, …, E. R. S. Kunji. 2015. Trends in thermostability provide information on the nature of substrate, inhibitor, and lipid interactions with mitochondrial carriers. *J. Biol. Chem.* 290:8206–8217.
5. Holzmann, N., C. Chipot, …, F. Dehez. 2016. Assessing the physiological relevance of alternate architectures of the p7 protein of hepatitis C virus in different environments. *Bioorg. Med. Chem.* 24:4920–4927.
6. Warschawski, D. E. 2015. Membrane Proteins of Known Structure Determined by NMR. http://www.drorlist.com/nmr/MPNMR.html.
7. Oxenoid, K., and J. J. Chou. 2005. The structure of phospholamban pentamer reveals a channel-like architecture in membranes. *Proc. Natl. Acad. Sci. USA.* 102:10870–10875.
8. Berardi, M. J., W. M. Shih, …, J. J. Chou. 2011. Mitochondrial uncoupling protein 2 structure determined by NMR molecular fragment searching. *Nature.* 476:109–113.
9. OuYang, B., S. Xie, …, J. J. Chou. 2013. Unusual architecture of the p7 channel from hepatitis C virus. *Nature.* 498:521–525.
10. Pebay-Peyroula, E., C. Dahout-Gonzalez, …, G. Brandolin. 2003. Structure of mitochondrial ADP/ATP carrier in complex with carboxyatractyloside. *Nature.* 426:39–44.
11. Verardi, R., L. Shi, …, G. Veglia. 2011. Structural topology of phospholamban pentamer in lipid bilayers by a hybrid solution and solid-state NMR method. *Proc. Natl. Acad. Sci. USA.* 108:9101–9106.
12. Cook, G. A., and S. J. Opella. 2011. Secondary structure, dynamics, and architecture of the p7 membrane protein from hepatitis C virus by NMR spectroscopy. *Biochim. Biophys. Acta.* 1808:1448–1453.
13. Ruprecht, J. J., A. M. Hellawell, …, E. R. S. Kunji. 2014. Structures of yeast mitochondrial ADP/ATP carriers support a domain-based alternating-access transport mechanism. *Proc. Natl. Acad. Sci. USA.* 111:E426–E434.
14. Guo, Y., R. C. Kalathur, …, W. A. Hendrickson. 2015. Protein structure. Structure and activity of tryptophan-rich TSPO proteins. *Science.* 347:551–555.
15. King, M. S., M. Kerr, …, E. R. S. Kunji. 2016. Formation of a cytoplasmic salt bridge network in the matrix state is a fundamental step in the transport mechanism of the mitochondrial ADP/ATP carrier. *Biochim. Biophys. Acta.* 1857:14–22.
16. Zhao, L., S. Wang, …, J. J. Chou. 2016. Specific lipid binding of membrane proteins in detergent micelles characterized by NMR and molecular dynamics. *Biochemistry.* 55:5317–5320.
17. Klingenberg, M. 2009. Cardiolipin and mitochondrial carriers. *Biochim. Biophys. Acta.* 1788:2048–2058.
18. Falconi, M., G. Chillemi, …, A. Desideri. 2006. Structural dynamics of the mitochondrial ADP/ATP carrier revealed by molecular dynamics simulation studies. *Proteins.* 65:681–691.
19. Dehez, F., E. Pebay-Peyroula, and C. Chipot. 2008. Binding of ADP in the mitochondrial ADP/ATP carrier is driven by an electrostatic funnel. *J. Am. Chem. Soc.* 130:12725–12733.
20. Wang, Y., and E. Tajkhorshid. 2008. Electrostatic funneling of substrate in mitochondrial inner membrane carriers. *Proc. Natl. Acad. Sci. USA.* 105:9598–9603.
21. Johnston, J. M., S. Khalid, and M. S. P. Sansom. 2008. Conformational dynamics of the mitochondrial ADP/ATP carrier: a simulation study. *Mol. Membr. Biol.* 25:506–517.
22. Aguayo, D., F. D. González-Nilo, and C. Chipot. 2012. Insight into the properties of cardiolipin containing bilayers from molecular dynamics simulations, using a hybrid all-atom/united-atom force field. *J. Chem. Theory Comput.* 8:1765–1773.
23. Hedger, G., S. L. Rouse, …, M. S. P. Sansom. 2016. Lipid-loving ANTs: molecular simulations of cardiolipin interactions and the organization of the adenine nucleotide translocase in model mitochondrial membranes. *Biochemistry.* 55:6238–6249.
24. Alexandrov, A. I., M. Mileni, …, R. C. Stevens. 2008. Microscale fluorescent thermal stability assay for membrane proteins. *Structure.* 16:351–359.
25. Bamber, L., M. Harding, …, E. R. S. Kunji. 2006. Yeast mitochondrial ADP/ATP carriers are monomeric in detergents. *Proc. Natl. Acad. Sci. USA.* 103:16224–16229.
26. Brüschweiler, S., Q. Yang, …, J. J. Chou. 2015. Substrate-modulated ADP/ATP-transporter dynamics revealed by NMR relaxation dispersion. *Nat. Struct. Mol. Biol.* 22:636–641.
27. Vignais, P. V., P. M. Vignais, and G. Defaye. 1973. Adenosine diphosphate translocation in mitochondria. Nature of the receptor site for carboxyatractyloside (gummiferin). *Biochemistry.* 12:1508–1519.
28. Klingenberg, M., K. Grebe, and B. Scherer. 1975. The binding of atractylate and carboxy-atractylate to mitochondria. *Eur. J. Biochem.* 52:351–363.
29. Krämer, R., and M. Klingenberg. 1977. Reconstitution of inhibitor binding properties of the isolated adenosine 5′-diphosphate,adenosine 5′-triphosphate carrier-linked binding protein. *Biochemistry.* 16:4954–4961.
30. Krämer, R. 1983. Interaction of membrane surface charges with the reconstituted ADP/ATP-carrier from mitochondria. *Biochim. Biophys. Acta.* 735:145–159.
31. Babot, M., C. Blancard, …, V. Trézéguet. 2012. Mitochondrial ADP/ATP carrier: preventing conformational changes by point mutations inactivates nucleotide transport activity. *Biochemistry.* 51:7348–7356.
32. Babot, M., C. Blancard, …, V. Trézéguet. 2016. Correction to Mitochondrial ADP/ATP carrier: preventing conformational changes by point mutations inactivates nucleotide transport activity. *Biochemistry.* 55:2422.

Biophysical Journal, Volume *113*

Supplemental Information

Mitochondrial ADP/ATP Carrier in Dodecylphosphocholine Binds Cardiolipins with Non-native Affinity

François Dehez, Paul Schanda, Martin S. King, Edmund R.S. Kunji, and Christophe Chipot

Mitochondrial ADP/ATP carrier in dodecylphosphocholine binds cardiolipins with non-native affinity

Supplementary material

François Dehez,[1] Paul Schanda,[2,*] Martin S. King,[3]
Edmund R. S. Kunji,[3] Christophe Chipot[1,4,*]

[1] Laboratoire International Associé Centre National de la Recherche Scientifique et University of Illinois at Urbana-Champaign, Unité Mixte de Recherche n°7565, Université de Lorraine, B.P. 70239, 54506 Vandœuvre-lè-Nancy cedex, France, [2] Institut de Biologie Structurale, CEA–CNRS–Université Grenoble Alpes, 71, avenue des Martyrs, C.S. 10090, 38044 Grenoble Cedex 9, France, [3] Medical Research Council, Mitochondrial Biology Unit, University of Cambridge, Cambridge Biomedical Campus, Wellcome Trust/MRC Building, Hills Road, Cambridge, CB2 0XY, United Kingdom, [4] Department of Physics, University of Illinois at Urbana-Champaign, 1110 West Green Street, Urbana, Illinois 61801

Thermal–shift assays. AAC3 was purified as described for the related yeast mitochondrial ADP/ATP carrier AAC2 (1). The purified AAC3 was diluted 20-fold in buffer containing 0.1% dodecyl maltoside (DDM) or 0.1% dodecylphosphocholine (DPC) and analyzed by thermal-shift assays, as described in reference 1. Protein unfolding is monitored with the maleimide coumarin fluorophore 7-diethylamino-3-(4'-maleimidylphenyl)-4-methylcoumarin (CPM). CPM reacts with protein thiols (4 cysteine residues in yAAC3) to give a fluorescent adduct, as cysteines become solvent-exposed due to denaturation of the protein. Thermal unfolding analysis was also performed using dye-free differential scanning fluorimetry (nanoDSF), which monitors the variations in fluorescence due to changes in the environment of 3 tryptophan and 11 tyrosine residues in yAAC3. Approximately 2 μg of protein at a concentration of 1.5 μM was added into a final volume of 10 μL buffer B (20 mM HEPES pH 8.0, 100 mM NaCl) containing either 0.1% of DDM with 0.1 mg/ml of tetraoleoyl cardiolipin, or 0.1% of DPC with 0.1 mg/ml tetraoleoyl cardiolipin, with or without 10 μM carboxyatractyloside, and the samples loaded into nanoDSF-grade standard glass capillaries. The temperature was increased by 5°C/min from 20 to 95°C, and the intrinsic fluorescence measured in a Prometheus NT.48 nanoDSF device (NanoTemper Technologies).

Simulation assays. For the simulation in a membrane mimetic, the molecular assembly consisted of yeast mitochondrial ADP/ ATP carrier yAAC3 embedded in a pre–equilibrated patch of 1–palmitoyl–2–oleo-yl–*sn*–glycero–3–phosphatidylcholine (POPC) with 18:2,18:2–cardiolipins (CLs), specifically tetralinoleyl–*sn*–glycero–*bis*–3–diphosphatidylglycerol (TLCL), consistent with the choice of lipids of Zhao et al. (2). Use was made of the crystallographic structure of yAAC3 obtained by Ruprecht et al. (3). Missing elements, in particular the N–terminus residues and one loop, were inferred using the program MODELLER (4). The lipid bilayer was formed by 124 POPC units and three CLs hydrated by 10,904 TIP3P(5) water molecules. After proper equilibration, the dimensions of the assay were approximately 74 $\times$ 74 $\times$ 98 Å^3. The molecular dynamics (MD) simulations were performed with a salinity of 0.15 M NaCl. The simulation assay was constructed employing the CHARMM–GUI membrane builder (6), which supplies randomized acyl–chain conformations for all lipid components. For the simulation in a micellar environment, yAAC3 binding the three CLs was embedded in DPC micelle with a detergent concentration of 300 nM, and a protein:detergent ratio of 1:250. After proper equilibration, the dimensions of the assay were approximately 103 $\times$ 103 $\times$ 103 Å^3. The all–atom CHARMM C36 force field (7) was used to describe water, yAAC3, the lipid bilayer (8) and the ions.

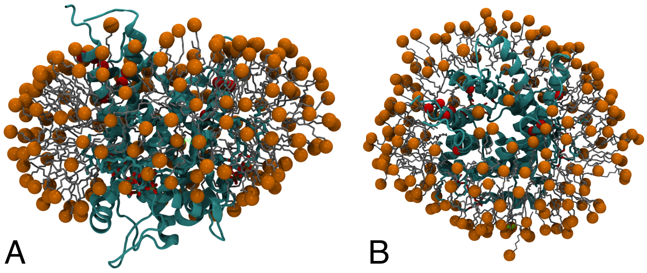

FIGURE S1: yAAC3 binding CLs embedded in a DPC micelle at a detergent concentration of 300 nM. (A) Side view and (B) top view.

© 2016 Biophysical Society
0006-3495/08/09/2624/12 $2.00

doi:

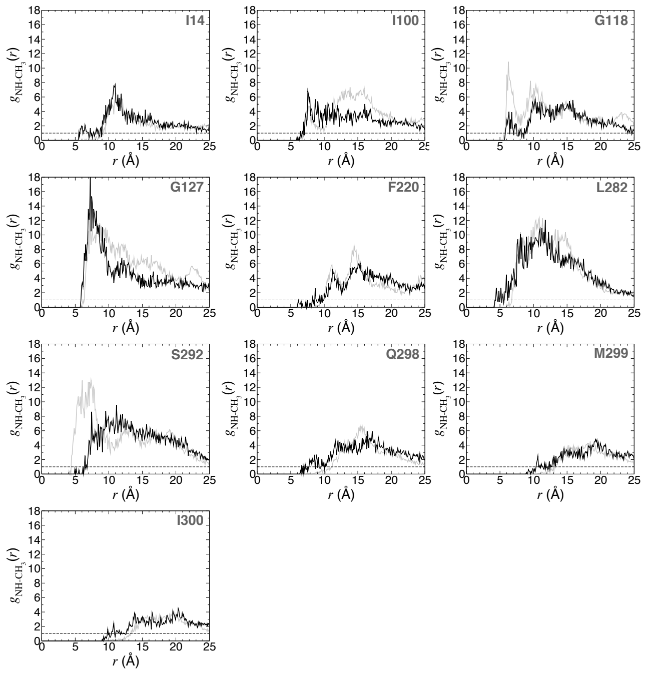

FIGURE S2: **Pair correlation functions between the –NH hydrogen atom of I14, I100, G118, G127, F220, L282, S292, Q298, M299 and I300, for which NOE effects were recorded by Zhao et al. (2) and the four terminal methyl groups of the three CLs bound to the mitochondrial carrier. The pair correlation functions were inferred from simulations in a POPC bilayer (dark lines) and in a DPC micelle (light lines).**

Molecular dynamics simulations. All simulations were performed in the isobaric–isothermal ensemble, using the scalable MD program NAMD 2.12 (9). Consistent with Zhao et al. (2), The temperature and the pressure were maintained at 300 K and 1 atm, respectively, using Langevin dynamics and the Langevin piston method, with anisotropic scaling of the simulation cell for the POPC assay (10). The r–RESPA multiple time–step algorithm(11) was employed to integrate the equations of motion with a time step of 1 and 2 fs for short– and long–range interactions, respectively. Covalent bonds in yAAC3 and in the lipids involving hydrogen atoms were constrained to their equilibrium length by means of the SHAKE/RATTLE algorithms (12, 13), and the SETTLE algorithms (14) for water. Long–range electrostatic forces were taken into account by means of the particle mesh Ewald algorithm (15) A 12–Å cutoff was introduced to truncate van der Waals and short–range Coulombic interactions. A switching function was introduced for van der Waals forces (8). Periodic boundary conditions were applied in the three directions of Cartesian space. The simulation protocols consisted of the following steps. For yAAC3 in a POPC bilayer, (i) after suitable energy minimization, the simulation assay was thermalized over 10.0 ns, during which both the protein and the CL head groups were harmonically tethered to their initial position, thereby allowing the lipid acyl chains to relax around the mitochondrial carrier. (ii) The assay was submitted to an additional 10.0–ns equilibration step, during which the positional harmonic restraints were removed and the entire molecular assembly was free to relax. (iii) A 200.0–ns production run was performed, from which configurations were stored every 10 ps for analysis purposes. For yAAC3 in a micellar environment, (i) after suitable energy minimization, the simulation assay was thermalized over 50.0 ns, during which both the protein and the CL head groups were harmonically tethered to their initial position, allowing the DPC units to optimize their interaction with the membrane carrier. (ii) A 100–ns production run was performed, from which configurations were stored every 10 ps for analysis purposes. Visualization and analyses of the MD trajectories were performed with the VMD program.(16)

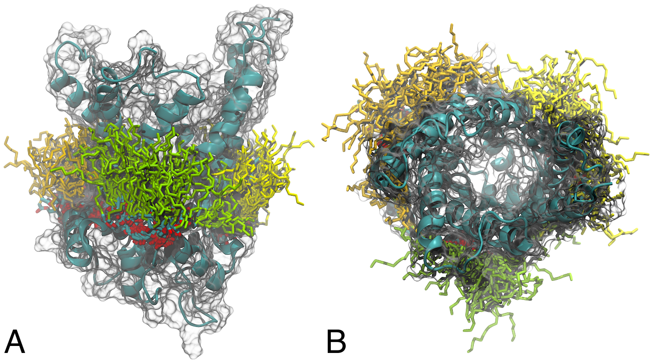

FIGURE S3: **Overlay onto the yAAC3 structure of CL conformations extracted every 10 ns from the 200–ns MD trajectory. (A) Side view and (B) top view. The superimposition of different conformations of the CLs underscores that while the head-group region remains tightly bound to the mitochondrial carrier in a specific fashion, the position of the acyl chains fluctuates markedly, indicative of a lack of specificity towards the protein. This representation further reinforces the idea that the CL acyl chains are highly unlikely to span the two leaflets of the lipid bilayer to interact with residues lining the mouth of the carrier, e.g., I300.**

NOE distances. In addition to the series of distances measured between the nitrogen atom of two amino acids, namely I14 and L282, and the terminal methyl group of the three CLs, and reported in the main text, a more systematic analysis was performed, wherein pair correlation functions (17) were determined for all the residues reported by Zhao et al. (see Figure S4 of reference 2), i.e., I14, I100, G118, G127, F220, L282, S292, Q298, M299 and I300. As can be seen in Figure S2, the computed pair correlation functions are strikingly at variance with the distances of Zhao et al. Consistent with Figure 3 of the main text, they reflect disorder and mobility of the CL acyl chains, as can be expected from thermal motion at 310 K, and the virtual impossibility of specific binding motifs with the mitochondrial carrier (see Figure S3).

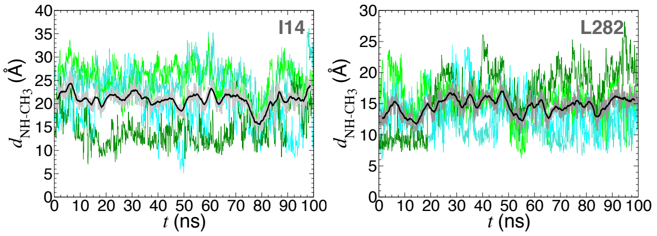

FIGURE S4: Distance separating the –NH hydrogen atom of residues I14 (left) and L282 (right) from the four terminal methyl group of the closest CL, as measured from the DPC assay. The four distances are shown as cyan, turquoise, light and dark green curves, alongside their mean as a thick grey curve and running average thereof as a black curve.

References

1. Crichton, P. G., Lee, Y., Ruprecht, J. J., Cerson, E., Thangaratnarajah, C., King, M. S., and Kunji, E. R. S. (2015) Trends in thermostability provide information on the nature of substrate, inhibitor, and lipid interactions with mitochondrial carriers. *J. Biol. Chem. 290*, 8206–8217.
2. Zhao, L., Wang, S., Run, C., OuYang, B., and Chou, J. J. (2016) Specific lipid binding of membrane proteins in detergent micelles characterized by NMR and molecular dynamics. *Biochemistry 55*, 5317–5320.
3. Ruprecht, J. J., Hellawell, A. M., Harding, M., Crichton, P. G., McCoy, A. J., and Kunji, E. R. S. (2014) Structures of yeast mitochondrial ADP/ATP carriers support a domain-based alternating–access transport mechanism. *Proc. Natl. Acad. Sci. U.S.A. 111*, E426–E434.
4. Webb, B., and Săli, A. (2014) Protein structure modeling with MODELLER. *Met. Mol. Biol.(Clifton, N.J.) 1137*, 1–15.
5. Jorgensen, W. L., Chandrasekhar, J., Madura, J. D., Impey, R. W., and Klein, M. L. (1983) Comparison of simple potential functions for simulating liquid water. *J. Chem. Phys. 79*, 926–935.
6. Jo, S., Lim, J. B., Klauda, J. B., and Im, W. (2009) CHARMM-GUI Membrane Builder for mixed bilayers and its application to yeast membranes. *Biophys. J. 97*, 50–58.
7. MacKerell Jr., A. D. et al. (1998) All–atom empirical potential for molecular modeling and dynamics studies of proteins. *J. Phys. Chem. B 102*, 3586–3616.
8. Klauda, J. B., Venable, R. M., Freites, J. A., O'Connor, J. W., Tobias, D. J., Mondragon-Ramirez, C., Vorobyov, I., MacKerell, A. D., Jr, and Pastor, R. W. (2010) Update of the CHARMM all–atom additive force field for lipids: Validation on six lipid types. *J. Phys. Chem. B 114*, 7830–7843.
9. Phillips, J. C., Braun, R., Wang, W., Gumbart, J., Tajkhorshid, E., Villa, E., Chipot, C., Skeel, L., R. D. Kalé, and Schulten, K. (2005) Scalable molecular dynamics with NAMD. *J. Comput. Chem. 26*, 1781–1802.
10. Feller, S. E., Zhang, Y. H., Pastor, R. W., and Brooks, B. R. (1995) Constant pressure molecular dynamics simulations — The Langevin piston method. *J. Chem. Phys. 103*, 4613–4621.
11. Tuckerman, M. E., Berne, B. J., and Martyna, G. J. (1992) Reversible multiple time scale molecular dynamics. *J. Phys. Chem. B 97*, 1990–2001.
12. Ryckaert, J., Ciccotti, G., and Berendsen, H. J. C. (1977) Numerical integration of the Cartesian equations of motion for a system with constraints: Molecular dynamics of n–alkanes. *J. Comput. Phys. 23*, 327–341.
13. Andersen, H. C. (1983) Rattle: a "velocity" version of the shake algorithm for molecular dynamics calculations. *J. Comput. Phys. 52*, 24–34.
14. Miyamoto, S., and Kollman, P. A. (1992) SETTLE: An analytical version of the SHAKE and RATTLE algorithms for rigid water models. *J. Comput. Chem. 13*, 952–962.
15. Darden, T. A., York, D. M., and Pedersen, L. G. (1993) Particle mesh Ewald: An $NlogN$ method for ewald sums in large systems. *J. Chem. Phys. 98*, 10089–10092.
16. Humphrey, W., Dalke, A., and Schulten, K. (1996) VMD — Visual molecular dynamics. *J. Molec. Graphics 14*, 33–38.
17. Chandler, D. *Introduction to modern statistical mechanics*; Oxford University Press, 1987.
